# Decoding Conformational Imprint of Convoluted Molecular Interactions Between Prenylflavonoids and Aggregated Amyloid-Beta42 Peptide Causing Alzheimer’s Disease

**DOI:** 10.3389/fchem.2021.753146

**Published:** 2021-12-20

**Authors:** E. Srinivasan, G. Chandrasekhar, P. Chandrasekar, K. Anbarasu, AS Vickram, Iftikhar Aslam Tayubi, R. Rajasekaran, Rohini Karunakaran

**Affiliations:** ^1^ Bioinformatics Lab, Department of Biotechnology, School of Bio Sciences and Technology, Vellore Institute of Technology (Deemed to be University), Vellore, India; ^2^ Department of Bioinformatics, Saveetha School of Engineering, Saveetha Institute of Medical and Technical Sciences, Chennai, India; ^3^ Department of Biotechnology, Saveetha School of Engineering, Saveetha Institute of Medical and Technical Sciences, Chennai, India; ^4^ Faculty of Computing and Information Technology, King Abdulaziz University, Jeddah, Saudi Arabia; ^5^ Unit of Biochemistry, Faculty of Medicine, AIMST University, Bedong, Malaysia

**Keywords:** alzheimer’s, amyloid-beta, neobavaisoflavone, herbal active compounds, computational screening

## Abstract

Protein misfolding occurs due to the loss of native protein structure and adopts an abnormal structure, wherein the misfolded proteins accumulate and form aggregates, which result in the formation of amyloid fibrils that are associated with neurodegenerative diseases. Amyloid beta (Aβ42) aggregation or amyloidosis is contemplated as a unique hallmark characteristic of Alzheimer’s disease (AD). Due to aberrant accrual and aggregation of Aβ42 in extracellular space, the formation of senile plaques is found in AD patients. These senile plaques occur usually in the cognitive and memory region of the brain, enfeebles neurodegeneration, hinders the signaling between synapse, and disrupts neuronal functioning. In recent years, herbal compounds are identified and characterized for their potential as Aβ42 inhibitors. Thus, understanding their structure and molecular mechanics can provide an incredible finding in AD therapeutics. To describe the structure-based molecular studies in the rational designing of drugs against amyloid fibrils, we examined various herbal compounds that belong to prenylflavonoids. The present study characterizes the trends we identified at molecular docking studies and dynamics simulation where we observed stronger binding orientation of bavachalcone, bavachin, and neobavaisoflavone with the amyloid-beta (Aβ42) fibril structure. Hence, we could postulate that these herbal compounds could be potential inhibitors of Aβ42 fibrils; these anti-aggregation agents need to be considered in treating AD.

## Introduction

**FIGURE 1:**
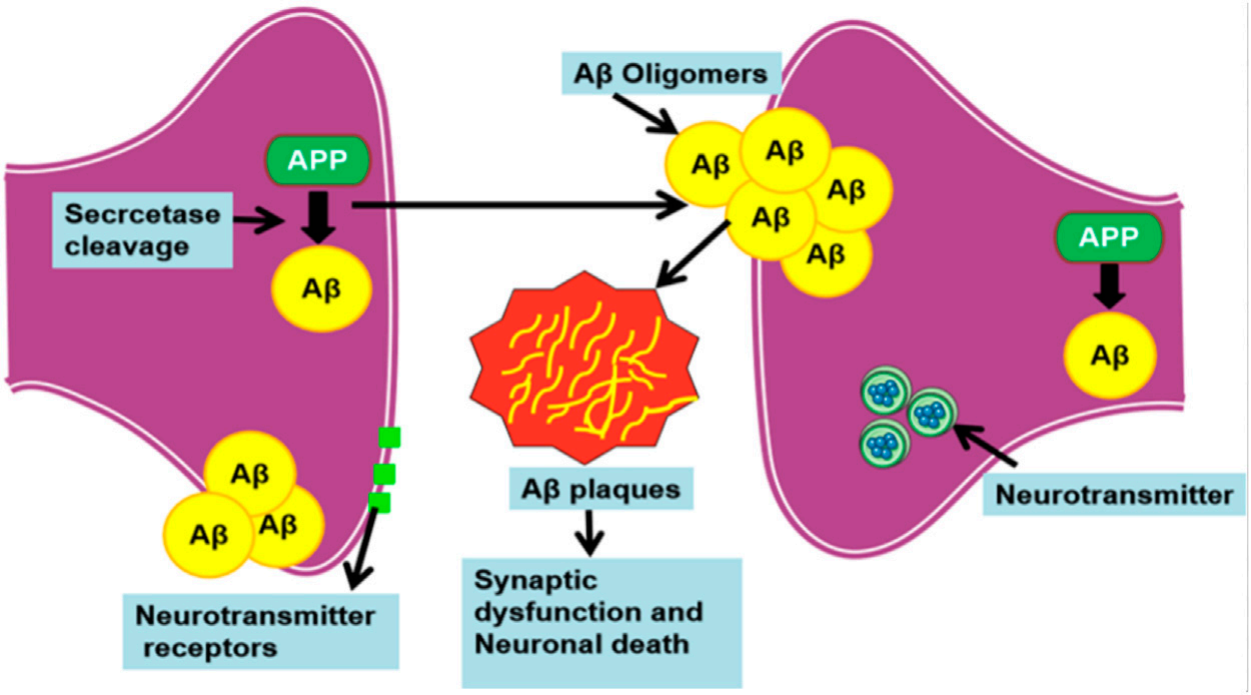
Process involved in the production of amyloid beta plaques.

Alzheimer’s disease (AD) is the archetypal impetus behind the mental deterioration of people over the age of 50, categorized as debilitative neurodegenerative disorder (Finder, 2010). Neuronal death, existence of neutrophil threads, specific loss of neurons, and synapse loss in the brain peculiarize AD. The essential key factors involved in the pathological prognosis of AD are the amyloid-beta (Aβ) peptide (rich in beta-sheet) constituted extracellular plaque formation, intracellular development of neurofibrillary tangles, and the degeneration of synapse (Al- Ayadhi et al., 2012; Gouras et al., 2015). Distinctly, beta-amyloid, an amyloid beta-peptide, is an aberrant protein that is increasingly noted for its protein misfolding activity in AD (Sarasa et al., 2000; Sweeney et al., 2017). In AD, amyloid beta-peptide 42 (Aβ42) is a well-known biomarker exhibiting the amyloidogenic activity that characterizes AD, due to the level of amyloid-beta deposits in cerebrospinal fluid. Secretases cleaves Aβ from large APP, thus producing the Aβ(1–42) to wrap up as an amyloid plaque that causes synaptic damage and neuron loss in brain (Obregon et al., 2012) (Figure 1). Aβ42 is known to be more neurotoxic because of the additional two amino acid residues called long-tailed, which leads to protein misfolding. Aβ42 is a 4-kDa soluble peptide (Owen et al., 1990; Murphy and LeVine, 2010), consisting of forty-two amino acids that resides inside the brain’s cortex. In particular, Aβ42 constitutes the majority of intraneuronal Aβ (Chen et al., 2017). These amyloid-beta oligomers form extracellular senile plaque deposits, protofibrils, and small oligomers with harmful effects such as synaptic damage, mitochondrial dysfunction, injury or dysfunction of neuronal cells, and death of neuronal cells that cause shrinkage and functional changes in the brain. This process is known as Amyloid cascade hypothesis, a hallmark in AD (Pimplikar, 2009; Reitz, 2012; Ricciarelli and Fedele, 2017). Specifically, the mutations such as KM670/671NL, A673T, A673V, D678H, E682K, K687N, A692G, and M722K increase the level of Aβ42 that leads to protein misfolding and accrual in AD. Typically, these aberrant proteins are supposed to be degraded by the ubiquitin (Ub)–proteasome system (UPS) and chaperone-mediated autophagy (CMA) pathways; however, under obscure circumstances, amyloid eludes the proteolytic pathways and furthers its accumulation in neuronal cells (Ciechanover and Kwon, 2015).

Small molecules are easy to administer, inexpensive to make, and can effectively traverse the blood–brain barrier, more rapidly. Furthermore, studies have shown that naturally occurring polyphenolic compounds are known to effectively modulate pathological aggregates of various proteopathic proteins. Attributable to its functional group, polyphenols interact with neurotoxic amyloids to act as potent anti-aggregation agents (Freyssin et al., 2018). Particularly, polyphenolic prenylflavonoid compounds from *P. corylifoliaseeds* were found to be neuroprotective in nature (Kim Y. et al., 2016; Lee et al., 2016). Bavachalcone and bavachin regulate the amyloid-beta produced by BACE-1 enzyme (Shewmaker et al., 2011). Neobavaisoflavone has remarkable anti-inflammatory properties (Colvin et al., 2015). Lately, several computational studies have been carried out to efficiently observe, assess, and quantify the dynamic biomolecular interaction between therapeutic candidates (Gurevich and Gurevich, 2014; Bulfone et al., 2018) and proteopathic targets like amyloids (Srinivasan and Rajasekaran, 2016, 2017, 2018a, 2018b, 2019; Srinivasan et al., 2019).

Therefore, in the present study, these three herbal prenylflavonoids were evaluated computationally to investigate their anti-aggregation potency and binding efficacy with the help of quantum mechanics, molecular docking, and dynamic simulations.

## Methodology

### Enhancement of the Aβ42 and Prenylflavonoids’ Structural Geometry

**FIGURE 2:**
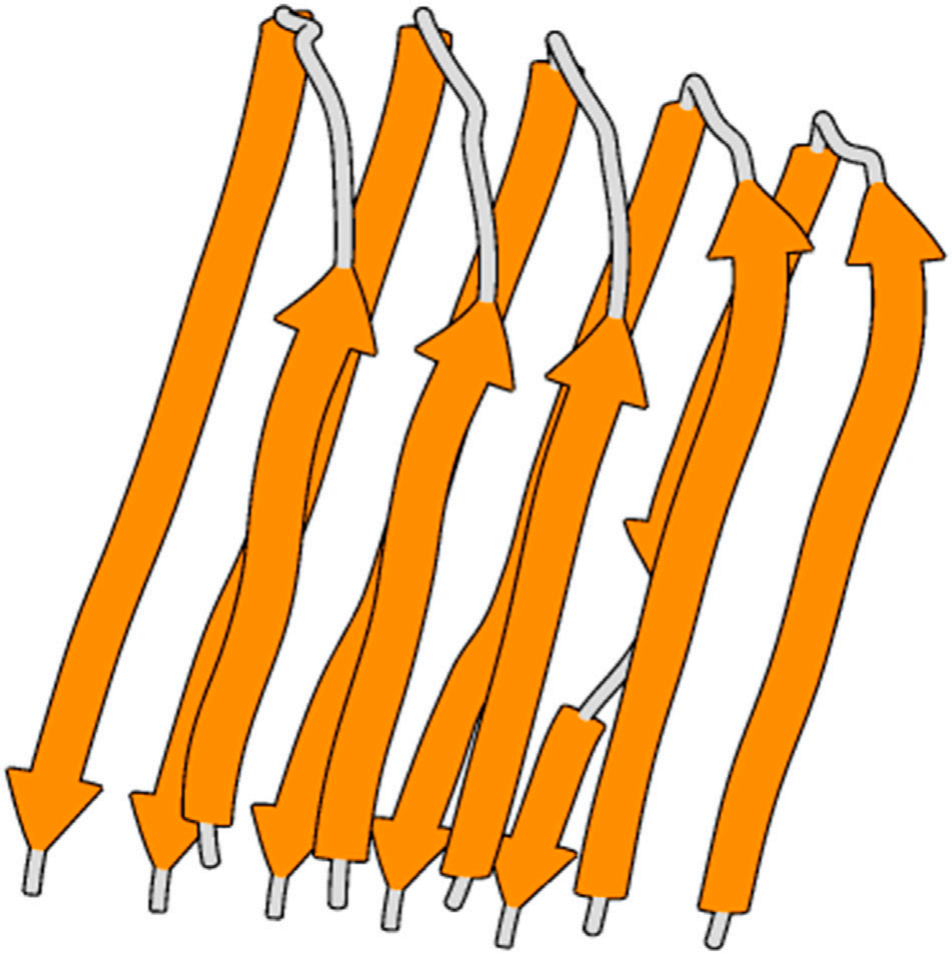
3D structure of amyloid-beta 42 fibrils.

Initially, the structural coordinates of native Aβ42 (Figure 2) were recovered from Protein Data Bank (PDB) (ID: 2BEG). To minimize the potential energy of the Aβ42 structural coordinates, GROningen MAchine for Chemical Simulations (GROMACS) with GROMOS 43a5 force field was employed (Hess et al., 2008). The molecular system was solvated within a cubic box with SPCE water molecules where the counter ions were added for neutralizing the system. Periodic boundary condition (PBC) and PME (Particle Mesh Ewald) were included in the simulation (Darden, York, and Pedersen., 1993). Finally, Steepest Descent algorithm was used to optimize the structural coordinates of Aβ42.

Small-molecule inhibitors were procured from PubChem, a proficient database that contains an extensive collection of chemical molecules (Kim S. et al., 2016). To optimize these structures, the def-SV(P) basis set containing TURBOMOLE’s B-3LYP functional set for DFT optimization was utilized (Steffen et al., 2010); these optimized structures were subjected to further analyses.

### Molecular Docking Studies

Using Autodock 4.2.3 software (Morris et al., 2009), docking simulation was performed, combining a fast energy assessment derived from pre-determined grids with separate search modules to find a suitable interaction location on the given protein for ligand. The structure being held tight during docking, the torsional bonds in small-molecule inhibitors were not restricted for flexible ligand docking. For docking calculations, we utilized the Lamarckian genetic algorithm with semi-empirical free energy and pre-computed grid maps. Using Auto Grid, we calculated the grid maps and used the default parameters to run the program. The grid maps were selected to encompass all amino acids with grid spacing between grid points set to 0.375 Å. Using Lamarckian Genetic Algorithm, we executed molecular docking operation to produce 100 potential complexes (of protein and ligand) by presenting ligand orientation search on each ligand 100 times for the protein model. We conducted triplicates of the ligand conformation search for protein for each ligand to procure the most accurate findings. The interface between ligand and protein was tested independently, using free energy computations that are ascertained semi-empirically. Besides, free energy was measured by adding intermolecular (van der Waals, hydrogen bond, electrostatic, and desolvation) energy, internal energy, torsional energy, and total energy for flexible ligand binding with protein. Furthermore, we examined the optimal docked complex with least binding energies.

### Ligand on Quantum Chemical Analysis

To be specific, we executed ligand quantum chemistry calculations, operating TURBOMOLE package DFT/B3LYP, raising single point energy measurement (Steffen et al., 2010). On the ground state, the ligands’ 3D structure was optimized completely. Initially, the input geometry was optimized, using the def-SV(P) basis set through DFT/B3LYP with the B3-LYP functional set for essential atoms like C, O, N, and H combining three Becke’s functional parameter exchange (B3) by Lee, Young, and Parr functional correlation (LYP).

### Discrete Molecular Dynamics

Furthermore, we executed structural dynamics through the simulation of DMD (Shirvanyants et al., 2012), the discrete molecular dynamics using distinct energy parameters computed with the discontinuous functions for calculating pairwise interaction; this study used the Atomistic Medusa DMD force field. Medusa force field is specifically parameterized for studying protein dynamics that effectively elucidates protein misfolding and illustrates conformational disturbances associated to mutations and other structural variations (Ding and Dokholyan, 2008). To elucidate a protein model exhibiting heavy atoms and polar hydrogen atoms, the united atom model was used. Covalent bonds, dihedrals, and bond angles comprise bonded interactions, while the environment-dependent H bonds, van der Waals, and solvation comprised bonded associations. The implicit Lazaridis–Karplus solvation model was used as a reference state to ascertain the solvated energy of conformations. With reaction-like algorithms, hydrogen bond interactions were modeled. With Debye–Huckel approximation, we modeled the screened charge–charge interactions by setting Debye duration to approximately 10 Å. Furthermore, distance restraints in between each metal atom and its commensurate metal-coordinating atoms were assigned for modeling the binding of metal ions, as stated from earlier studies. With a reaction algorithm, the distance and coordination dependency of the establishment of disulfide bonds were also modeled (Ding and Dokholyan, 2008). For performing DMD simulations, the volume and periodic boundary conditions were kept constant; we employed Anderson thermostat to regulate and maintain fixed temperature during simulation. Additionally, the configuration of the simulation system was snapshotted once in every 100 time units and the simulation was carried out for a time period of 1 × 10^5^ time units.

Herein, DMD simulations signify the length [L] in Angstrom (10^−10^ m), the time unit [T] as determined by the mass units [M] in Dalton (1.66 × 10^−24^ g), and energy [E] in kcal/mol (6.9 × 10^–22^ J). With respect to classical MD, approximately 50 fs represents each time unit (Ding and Dokholyan, 2008). Finally, geometrical assessment on all the structural trajectories enumerated throughout the simulation were performed, using GROMACS such as Define Secondary Structure of Proteins (DSSP) (secondary structural propensity), g-rms (conformational deviation), g-gyrate (protein gyration), and g-rmsf (conformational flexibility).

### Steered Molecular Dynamics

With Yet Another Scientific Artificial Reality Application (YASARA), SMD was carried out for bavachalcone, bavachin, and neobavaisoflavone attached to Aβ42 fibril and native Aβ42 fibril, keeping the temperature constant at 298 K. Furthermore, we conducted the simulations employing AMBER03 force field (Wang et al., 2004) in a solvation box of 0.997 g ml^−1^solvent density water molecules. In addition, we neutralized the system charge assigning 0.9% NaCl. Moreover, we maintained pH at 7.0 throughout the simulation. Together with periodic boundary conditions, long-range coulomb forces were added; thus, we assigned these parameters to perform energy minimization with steepest descent algorithm. In addition, we started the simulation by fixing the pulling acceleration to 1,000 pm/ps^2^ to independently extract bavachalcone, bavachin, and neobavaisoflavone compounds from the native complex, because the software uses constant acceleration to conduct SMD. However, the mass center of native and mutant Aβ42 was kept constant, and the complex was pulled in each direction. At a distance of 0.4 nm, SMD ended when the bavachalcone, bavachin, and neobavaisoflavone were completely unbound from the native structure, signifying that the complexes completely dissociated these herbal compounds. Subsequently, the simulation snapshots were saved at every 10-ps interval.

### Free Energy Landscape

To achieve the near-native structural conformation employing the conformational sampling process, we obtained the free energy protein landscape. At this point, DMD was performed to sample mutant and mutant-complex protein conformations. We used two critical components as reaction coordinates, viz., root-mean-square deviation (RMSD) and radius of gyration (Rg) to acquire free energy landscape. With these two components, we determined the energy landscape based on the following equation:

ΔG(p1,p2)=−kBT In (p1,p2)

Herein, kB depicts the Boltzmann constant, while ∆G denotes the Gibbs free energy of state, and T represents the temperature maintained during the of simulation. p1, p2 depicts the reaction coordinates, which is used to construct 2D landscape based on the joint probability distributions: P (p1, p2) obtained from the system (Papaleo et al., 2009).

## Results and Discussion

### Protein–Ligand Binding and Interaction Analysis

**FIGURE 3:**
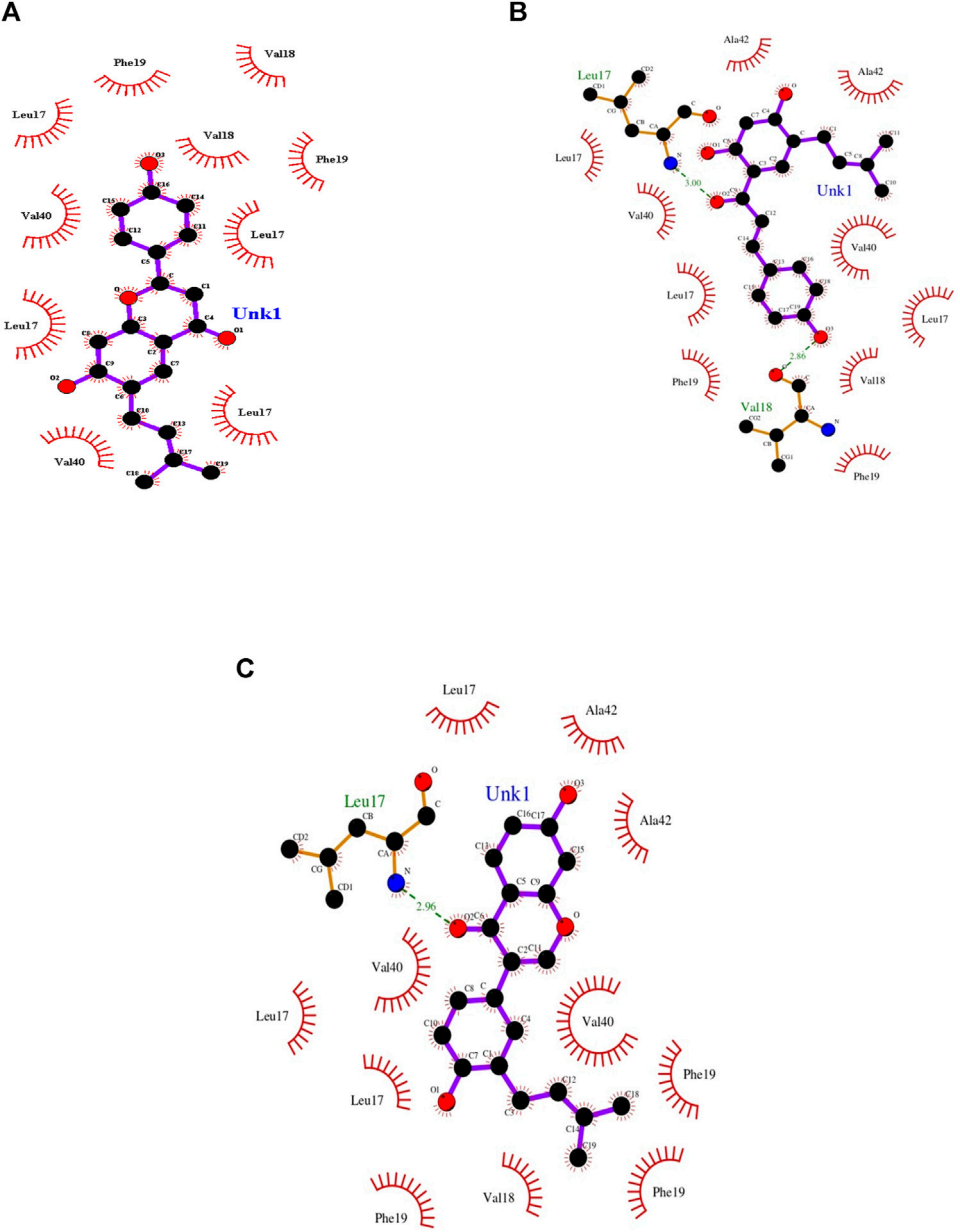
Docking view of Aβ42 fibril structure interacting with the three best docked herbal compounds **(A)** bavachin, **(B)** bavachacone, and **(C)** neobavaisoflavone that forms hydrogen bond (brown) and hydrophobic residues (orange) were plotted using LIGPLOT.

Docking-optimized structures of both the receptor and ligand were created to identify molecules that may bind to the interested protein target (Sethi et al., 2019). Molecular docking is an essential aspect in the case of structure-based drug designing. Docking strategies explore high-dimensional spaces effectively, and the scoring functions are used to rank the small-molecule candidates that are docked with the receptor protein. Moreover, the molecular docking process evaluates a protein–receptor complex using various factors such as binding energies and intermolecular interactions that include hydrogen bonds and hydrophobic interactions (Meng et al., 2011; Leelananda and Lindert, 2016; Wang and Zhu, 2016). From those mentioned above, the more potent, selective, and efficient drug candidates could be developed to treat AD. The NMR structure of Aβ42 (2BEG) from PDB is used as a protein receptor. The compounds are geometrically optimized and the water molecules are detached for performing molecular docking studies. Consequently, the herbal compounds, bavachalcone, bavachin, and neobavaisoflavone were embedded in the active site of the Aβ42 receptor as the best docked complex based on binding orientation (Table 1). Thus, the docked complex of Aβ42-bavachalcone exhibits about −8.23 kcal/mol binding energy, while bavachin and neobavaisoflavone exhibited −8.10 and −8.09 kcal/mol binding energy, respectively. From the results, we could infer that bavachalcone has a higher binding energy comparatively than bavachin and neobavaisoflavone. During the interactions, a bavachalcone molecule forms two hydrogen bonds with residues Leu17 (chain A) and Val18 (chain C) amyloid-beta fibril. The distance of the hydrogen bond for Leu17 is 3.00 Å, and that for Val18 is 2.86 Å. The bavachin compound does not form a hydrogen bond with the Aβ42 receptor. A neobavaisoflavone molecule forms one hydrogen bond with residue Leu17 of chain C at a distance of 2.96 Å (Figure 3) (Table 2). Comparatively, neobavaisoflavone has a lower binding energy, but it forms a hydrogen bond with the receptor, which is essential in forming a stabilized protein–ligand complex. In accordance, bavachalcone and neobavaisoflavone are considered to be more effective inhibiting complexes. However, the hydrophobic effect is essential in the arrangement of Aβ42 oligomers into stabilized Aβ42 fibrils. Thus, the hydrophobic residues demonstrate the stabilization of amyloid-beta fibril (Marshall et al., 2011). Interaction of bavachalcone and neobavaisoflavone with Aβ42 fibril structure binds the hydrophobic residues Leu17, Val40, Phe19, Val18, and Ala42. Bavachin forms the hydrophobic interactions with Leu17, Val40, Phe19, and Val18 **(** Figure 3). Hydrophobic interactions of both bavachalcone and neobavaisoflavone molecules have common amino acid residues, and therefore, the selected herbal compounds have shown considerable binding energy.

**TABLE 1:** Compounds utilized for the molecular docking with amyloid-beta peptide.

No	Compound name	Ligand structure	Iupac name	Chemical formula	Molecular weight
1	Bavachalcone	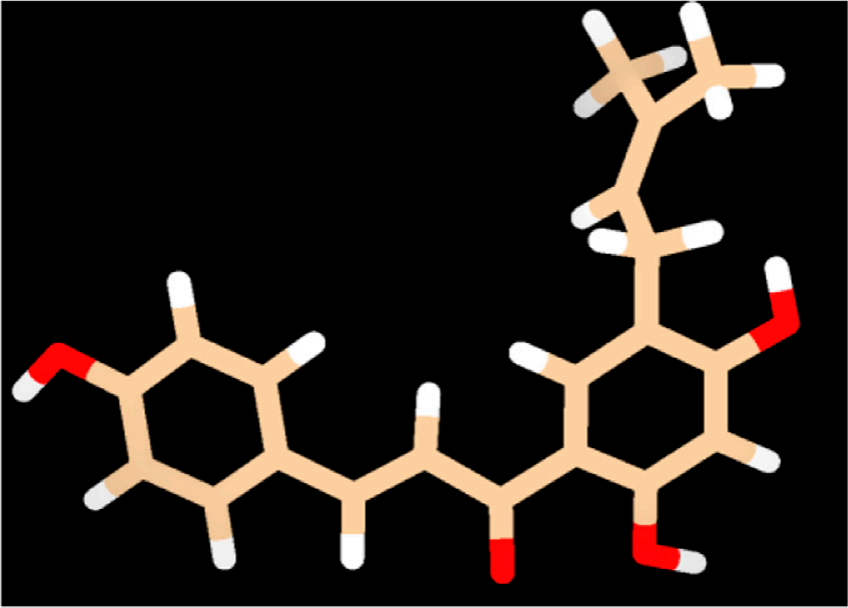	(E)-1-[2,4-dihydroxy-5-(3-methylbut-2-enyl)phenyl]-3 (4hydroxyphenyl) prop-2-en-1-one	C_20_H_20_O_4_	324.4 g/mol
2	Bavachin	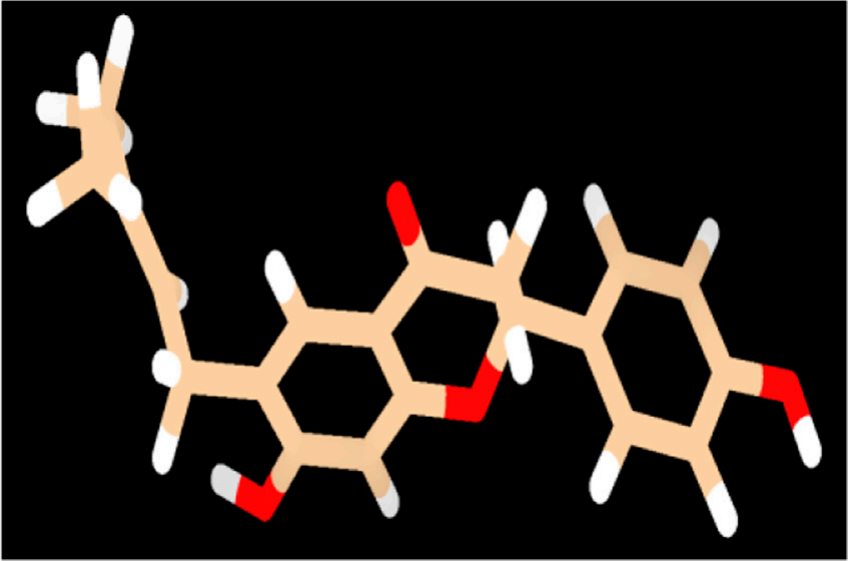	(2S)-7-hydroxy-2-(4-hydroxyphenyl)-6-(3-methylbut-2-enyl)-2,3-dihydrochromen-4-one	C_20_H_20_O_4_	324.4 g/mol
3	Neobavaisoflavone	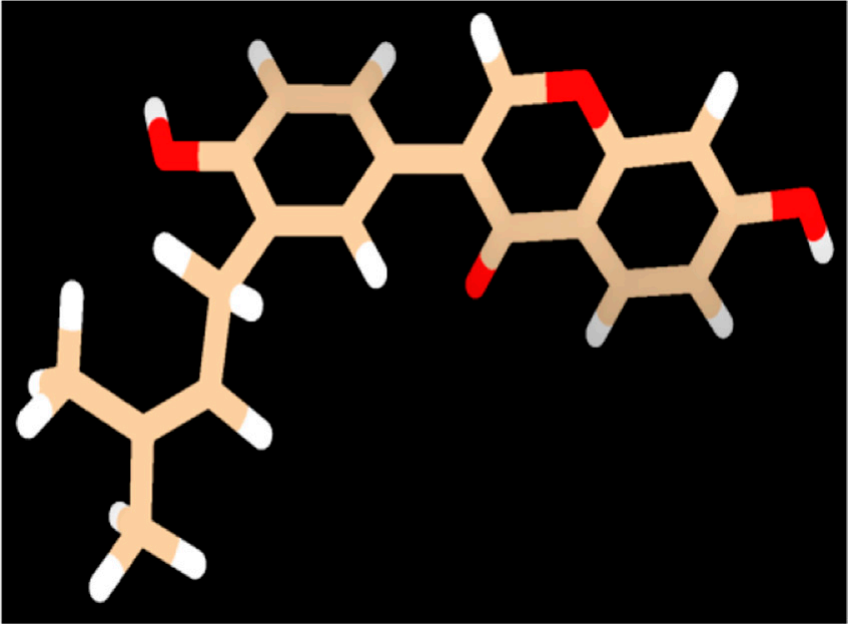	7-hydroxy-3-[4-hydroxy-3-(3-methylbut-2-enyl)phenyl]chromen-4-one	C_20_H_18_O_4_	322.4 g/mol

**TABLE 2:** Interactions resulted from docking analysis of the compounds with amyloid-beta peptide.

Docked compound	Binding energy (kcal/mol)	Hydrogen bonds	Hydrophobic interactions
Bavachalcone	−8.23	Leu17, Val18	Leu17, Val40, Phe19, Val18, Ala42
Bavachin	−8.10	—	Leu17, Val40, Phe19, Val18
Neobavaisoflavone	−8.09	Leu17	Leu17, Val40, Phe19, Val18, Ala42

To further investigate into the protein–ligand associations, the prenylflavonoids complexes’ dissociation constants were calculated upon binding. Herein, epigallocatechin gallate (EGCG), an evinced anti-amyloid that showed potency at minimal concentrations of 7.5 mg/ml (Lee et al., 2009), was used as a positive control to compare the dissociation constant metrics with the prenylflavonoids being analyzed. Reaction kinetics states that the lower the dissociation constant, the higher the binding affinity between the protein and ligand (Corzo, 2006; Du et al., 2016); accordingly, calculations show that all the three prenylflavonoids’ dissociation constants were on par with each other with slight variations, and more importantly, the values were substantially lower when compared with EGCG’s dissociation constant upon binding with Ab42 amyloid fibril (Table 3). Findings indicate that compared to positive control EGCG, all the three prenylflavonoids evince a considerably higher interaction with Ab42 amyloid fibril. Hence, combining docking scores and dissociation constant values, bavachalcone and neobavaisoflavone reported a higher intermolecular interaction than bavachin complex, which insinuates a potential alleviation of Ab42 mediated pathology, since the higher the binding, the better the ligand pose holds on the protein (Kumar and Doss, 2016).

**TABLE 3:** Table elucidating dissociation constant of Ab42 peptide complexed with ligands.

Ab42 complexed with ligands	Dissociation constant (microMolar)
Bavachalcone	0.99
Bavachin	1.14
Neobavaisoflavone	1.18
EGCG	40.19

**FIGURE 4:**
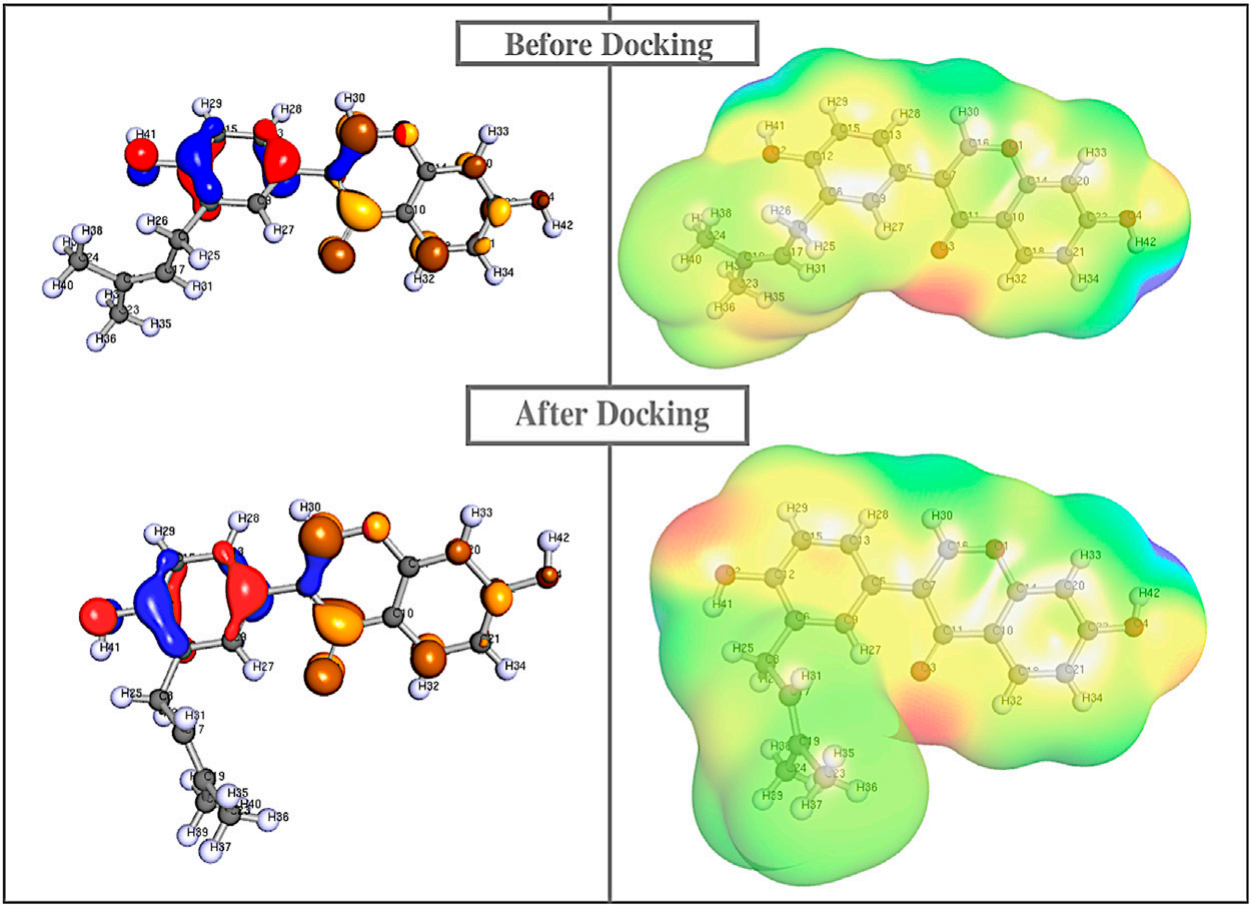
Quantum mechanics on all the compounds before and after docking were calculared. The HOMO (red/blue), LUMO (brown/yellow), and electrostatic potential were computed using Def-SVP basis set with B3-LYP function in which Neobavaisoflavonealone exhibited greater change in the energy gap between the apo and docked complex state.

To provide a detailed understanding on the structural interaction, we performed atomic-level studies on the compound structures recovered before and after docking, using the quantum mechanics tool. Conversely, findings from the QM analysis exposing the HOMO/LUMO energy gap difference of the compounds, before and after docking (Table 4), suggested that neobavaisoflavone showed a considerable variation in the energy gap in contrast to that of other compounds (Figure 4). To further substantiate the static analysis from the molecular docking and quantum mechanics studies, we utilized discrete molecular dynamics to illustrate the association of compounds over aggregated Aβ42protein through dynamic scale within a defined system over a period of time.

**TABLE 4:** HOMO/LUMO energy gap of the compounds before and after docking.

Compounds	HOMO-LUMO energy gap (eV)		EnergyDifference (eV)
	Before docking	After docking	
Bavachalcone	4.052	4.291	−0.239
Bavachin	4.766	4.665	0.111
Neobavaisoflavone	4.19	3.866	0.324

### Discrete Molecular Dynamic Simulations Protein–Ligand Complex and Native

**FIGURE 5:**
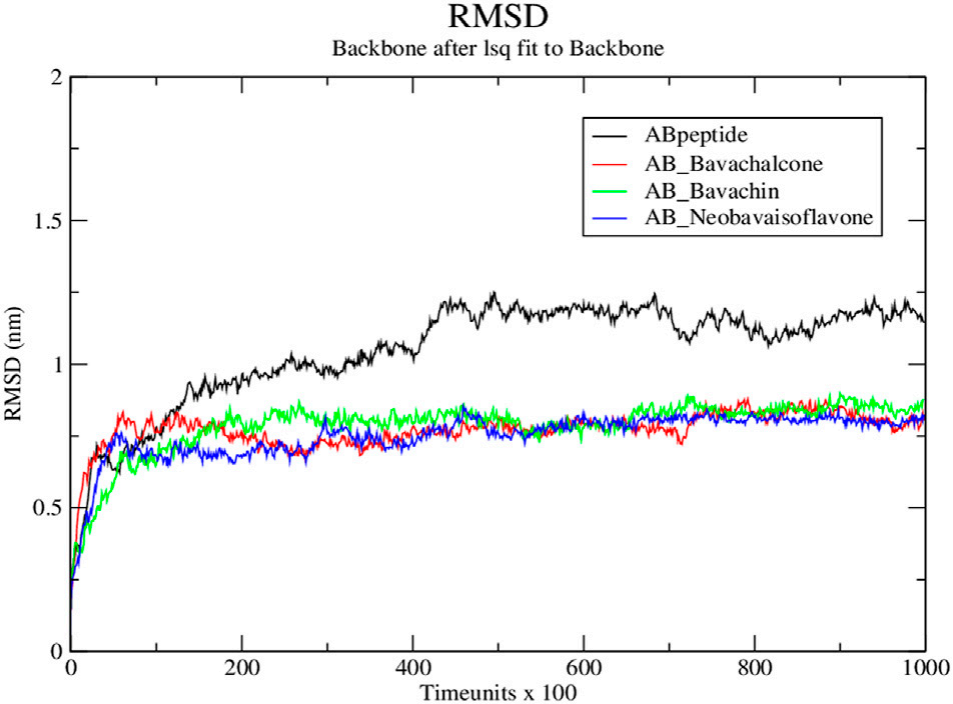
RMSD graph for protein–ligand complex from Discrete molecular dynamics.

Subsequently, we simulated the above-mentioned docked compounds for 1 × 10^5^ time units, wherein root mean square deviation (RMSD) was measured by plotting RMSD versus time. In native simulations, RMSD values are rising rapidly and attain the stability at 0.8 nm; the protein–ligand complex showed fluctuations, stabilized at approximately 1.2 nm, and then, it was rather steady during the rest of simulation. Thus, the protein–ligand complex was found to stabilize during the simulation (Kato et al., 2017). Based on the molecular simulation, RMSD values of bavachalcone, bavachin, and neobavaisoflavone bound to Aβ42 fibril were stabilized between 0.5 and 1 nm, thereby maintaining the flexibility and compactness. There were no fluctuations found in protein–ligand complexes, while the native amyloid fibril showed a more significant deviation at a range of 1–1.3 nm (Figure 5). Correspondingly, these three compounds exhibited stronger binding to Aβ42 fibril, due to the compact and stable structure.

**FIGURE 6:**
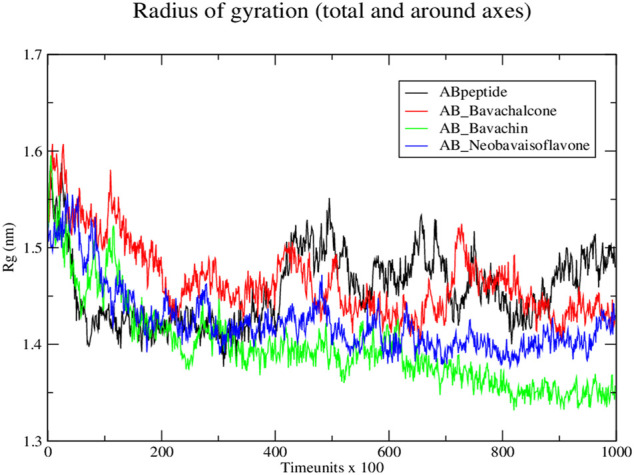
Radius of gyration graph for protein–ligand complex from Discrete molecular dynamics.

**FIGURE 7:**
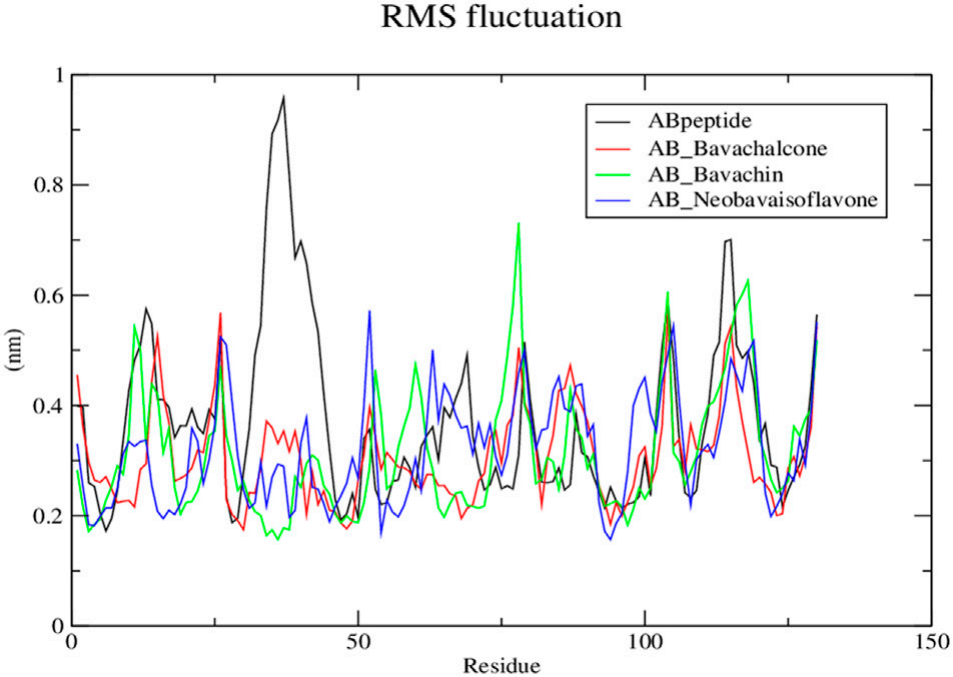
Root mean square fluctuation graph for protein–ligand complex from Discrete molecular dynamics.

Subsequently, the protein–ligand complex stability was further analyzed, using Rg. Amyloid fibril native displayed a Rg value of approximately 1.5 nm; the fluctuation decreased with time and formed a stable structure at 1.55 nm, whereas both bavachalcone and bavachin complex displayed a Rg value of 1.6 nm that decreased with time and stabilized at 1.49 and 1.35 nm, thereby exhibiting a greater difference in their compactness with Aβ42 fibril, respectively. However, neobavaisoflavone bound with Aβ42 fibril with a Rg value of 1.5 nm, which further decreased and stabilized at approximately 1.44 nm (Figure 6), during the complete course of simulation. In comparison to the Rg value of native Aβ42 fibril, the protein–ligand complexes displayed the lowest Rg that was stabilized, thus suggesting the lower Rg value resulting in tight bonding of the resultant complex. Thus, in the course of the simulation, Aβ42 binding with bavachalcone, bavachin, and neobavaisoflavone complex was found to be more compact and stable. Furthermore, RMSF analysis was further evaluated to determine the stability and flexibility of the complexes, where the fluctuations were observed, during the process of bavachalcone, bavachin, and neobavaisoflavone binding to the surface of Aβ42 fibril (Figure 7). RMSF values validate that neobavaisoflavone was more effective in binding to the hydrophobic core of Aβ42 compared to bavachalcone and bavachin. Thus, the interaction of Aβ42 fibril with bavachalcone, bavachin, and neobavaisoflavone complexes demonstrated the inhibitory effects to destabilize the fibril structure and prevent plaque formation. From the herbal compounds, we examined that due to the loss of hydrogen bond interaction, bavachin is not found to be impressive. Resultantly, neobavaisoflavone is found to be more effective in inhibitory actions, thereby showing a stronger

### SMD Evaluation

**FIGURE 8:**
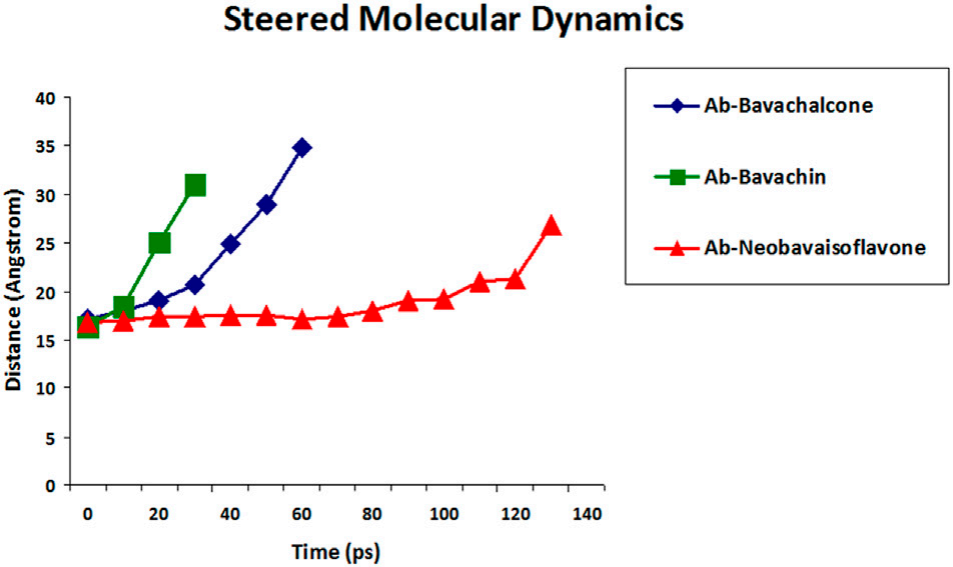
SMD was performed to measure the unbinding period of compounds bavachalcone (blue), bavachin (green), and neobavaisoflavone (red) that are bound to Aβ42 fibril.

To understand the molecular structure of a compound, the association properties such as hydrogen bonds, hydrophobic residues, and the dissociation properties are essential. Steered molecular dynamics plays a vital role in the field of drug designing to measure its stability and in studying the relationship between the protein–ligand complex (Do et al., 2018). The protein–ligand complex that unbinds increase in time (picoseconds) has strong binding, which is found to be a more stable complex. Thus, results from the SMD simulations showed the time required to disassociate bavachalcone (60 ps), bavachin (30 ps), and neobavaisoflavone (130 ps) from Aβ42 fibril (Figure 8). Based on the results, we could clearly infer that neobavaisoflavone has greater binding strength and stability in contrast to that of bavachalcone and bavachin.

Furthermore, the binding efficacy of aforementioned polyphenols was compared with the metrics of proven potent anti-amyloid EGCG, which has exhibited considerable binding and anti-aggregate proclivities against Aβ42 fibril aggregates (Rezai-Zadeh et al., 2005; Park et al., 2020; Zhang et al., 2020). The higher the time taken during SMD, the higher the binding efficacy between the protein and ligand. Accordingly, the positive control EGCG evinced 50 ps to completely dissociate from amyloid Aβ42, which is comparable to that of bavachalcone and a bit more when compared with bavachin. However, SMD values of neobavaisoflavone is considerably higher when compared to EGCG, which indicates that the former shows notable interaction with the amyloid, which is significantly higher than the well-established positive control EGCG’s interaction with the same. Thus, the docking studies and the SMD simulations altogether conclude that neobavaisoflavone is found to be more stable as compared to that of other compounds, which narrowed down our study towards further analysis.

### Secondary Structure Studies

Interactions between the polypeptide chains containing the alpha-helix and beta-pleated sheets evince a crucial part in the formation of a secondary structural framework in a protein. H-bond formation among the residues leads to the evolution of alpha-helix and beta-pleated sheets. Therefore, secondary structure properties such as β-sheets, coil, turn, helix, and others are evaluated, which supports this study. Accordingly, the percentage of secondary structure values for bavachalcone, bavachin, and neobavaisoflavone of coil, turn, β-bridge, and alpha-helix was found to be increased in comparison to native (Table 5). From the overall results, neobavaisoflavone has drastic reduction in β-sheet propensity, with the increase in turns, coils, and helix indicating the degrading ability of the compound and preventing the formation of β-strands, followed by the formation of amyloid aggregates.

**TABLE 5:** Comparison of Aβ42 (native) secondary structure against bavachalcone, bavachin, and neobavaisoflavone using the DSSP program.

Secondary structure elements	Native	Bavachalcone	Bavachin	Neobavaisoflavone
Structure	53	40	44	35
Coil	32	43	40	47
β-Sheet	42	27	29	17
β-Bridge	4	4	5	7
Bend	12	14	11	14
Turn	7	7	8	8
Alpha-Helix	0	2	2	2
3-Helix	0	0	2	0

### FEL Analysis

**FIGURE 9:**
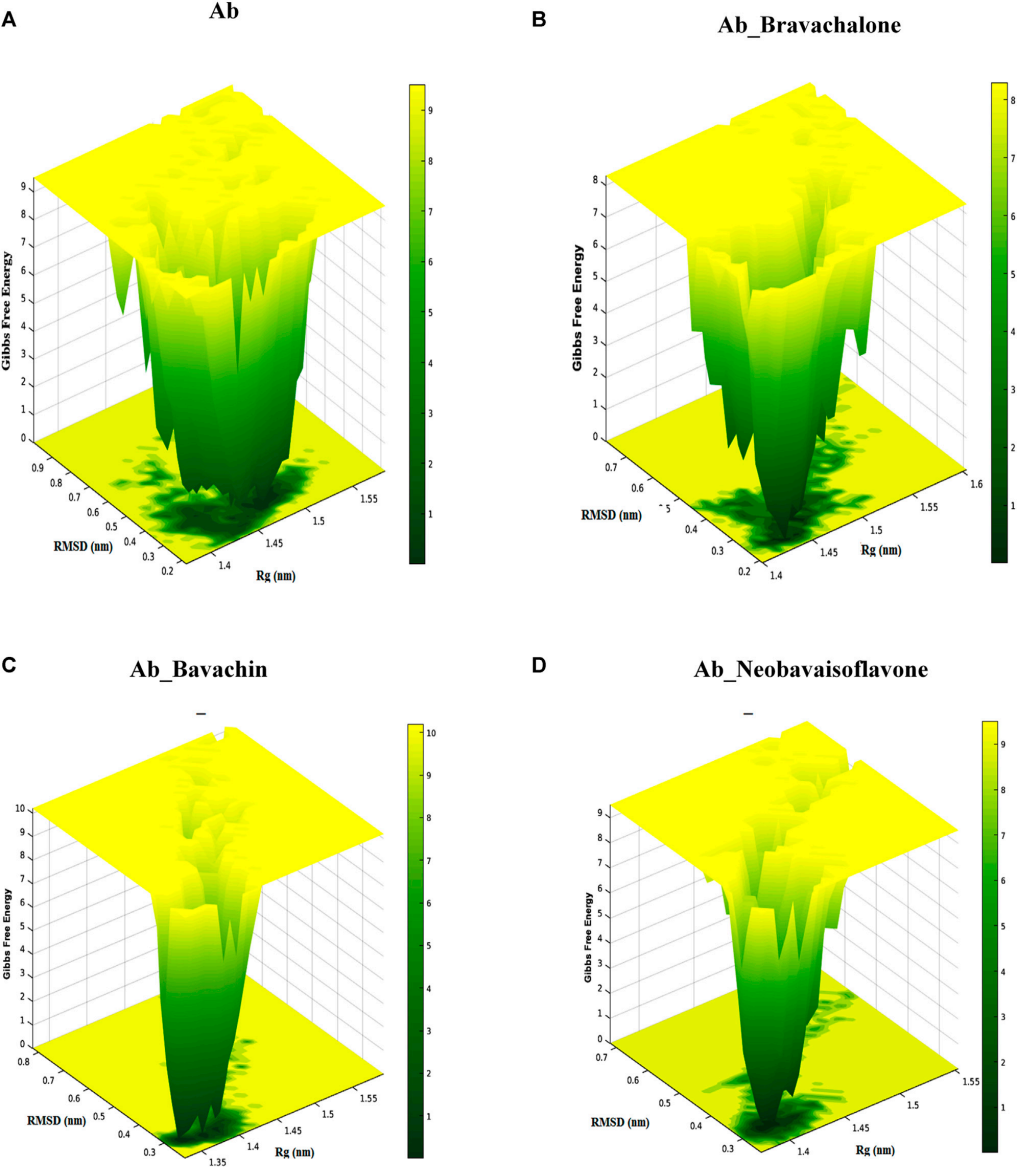
Free energy landscape presents the view of Aβ42 aggregates and Aβ42 aggregates obstructed by binding to bavachalcone, bavachin, and neobavaisoflavone. The study resulted in the compound neobavaisoflavone being quite efficient in inhibiting the formation of Aβ42 aggregates.

Free energy landscape (FEL) was evaluated to analyze the structural changes that support our study for understanding the destabilization of Aβ42 fibril. FEL represents the total number of interactions between residues and the number of interactions that correspond to the most stable native structure. Furthermore, the free energy landscape for the protein–ligand complex exhibited varying Gibbs free energy in between the range of 1–10 kcal/mol. Amyloid fibrils are typically polymorphic in nature, which is one of the chief characteristics of amyloids (Tycko, 2015; Close et al., 2018; Fändrich et al., 2018), and to further explore the pathogenic aspects of amyloid from this perspective, FEL was construed. We determined the FEL of Aβ42 fibril and its complex herbal compounds such as bavachalcone, bavachin, and neobavaisoflavone by utilizing the RMSD and Rg coordinates (Figure 9). The FEL for unbound Aβ42 fibril resulted in multiple free energy basins that are located within the Rg value of 1.47 nm and RMSD value of 0.3 nm, respectively. An increase in the development of multiple free energy basins indicates an increase in the formation of fibril structures that bind together and produce senile plaques (Wang et al., 2014). Bavachalcone bound to Aβ42 fibril forms two basins with Rg and RMSD values of 1.44–1.46 nm and 0.25–0.3 nm, respectively, whereas bavachin binding Aβ42 fibril forms three free energy basins between 1.3 and 1.4 nm Rg values and about 0.3 nm RMSD value. In contrast to free energy values of the above-mentioned compounds, neobavaisoflavone produced only one confirmative basin within a Rg value of 1.4 nm and a RMSD value of 0.3 nm, implying a conformational restriction by the polyphenol over Aβ42. A decrease in the number of free energy basins represents an increase in the inhibitory effect of the herbal compound. Though the compounds bavachalcone and bavachin have a reduction in the number of basins, neobavaisoflavone has the least number of only one basin, which is found to be more efficient comparatively. Therefore, the overall results obtained from molecular docking, molecular dynamics simulations, steered molecular dynamics, secondary structure analysis, and FEL analysis exposed that neobavaisoflavone has considerable binding strength with Aβ42 fibril and the ability to destabilize fibril formation, compared to bavachalcone and bavachin. Based on this study, the prenylflavonoid neobavaisoflavone could act as a potent therapeutic compound in advancing anti-Alzheimer’s drug development.

## Conclusion

Therapeutic agents for various diseases flooded in nature are still unidentified. Comparatively, the natural compounds are safe and show a reduced level of side effects than the chemical compound-based drug discovery. Therefore, the researchers take their steps in search of natural compounds for curative therapies. In recent years, the herbal compounds have been an essential component in AD treatment, wherein the mechanism of inhibition depends on the aromatic and hydrophobic association between the misfolded protein aggregate and the small-molecule inhibitors. Reduction in the accumulation of misfolded Aβ42 structures by stabilizing the native conformation can be used in the prevention of amyloid-beta aggregation. *In silico* approach of structure-based drug discovery can be used to identify herbal compounds that target amyloid-beta 42 aggregates. According to the docking results, bavachalcone, bavachin, and neobavaisoflavone have been identified as potent inhibitors with the highest binding affinity among the docked compounds along with the control EGCG. SMD and QM results reinforced neobavaisoflavone for exhibiting a strong binding effect compared to other compounds. Besides, the formation of a single free energy basin resulting from free energy landscape showed that neobavaisoflavone has a greater stability and abides by the properties of the drug compared to bavachalcone and bavachin. This approach is used for the identification of potent and specific drug lead compounds that break Aβ42 β-sheets using small molecules, thereby inhibiting and reversing Aβ42 misfolding and oligomerization activity. Hence, developing a structure-based drug design using the pharmacophore of naturally available prenylflavonoids could play a significant role in AD treatment.

## Data Availability

The original contributions presented in the study are included in the article. Further inquiries can be directed to the corresponding author.
